# Retraction Note to: LncRNA SNHG12 regulates the radiosensitivity of cervical cancer through the miR-148a/CDK1 pathway

**DOI:** 10.1186/s12935-022-02628-5

**Published:** 2022-06-09

**Authors:** Chen Wang, Shiqing Shao, Li Deng, Shelian Wang, Yongyan Zhang

**Affiliations:** 1 Department of Obstetrics and Gynecology, Huaihe Hospital of Henan University, No.8 Baogonghu North Road, KaifengHenan, 475000 China; 2 Department of Obstetrics and Gynecology, The Second Afliated Hospital of Guangxi Medical University, Nanning, Guangxi China

## Retraction to: Cancer Cell Int (2020) 20:554 10.1186/s12935-020-01654-5

The Editors-in-Chief have retracted the article because the authors have been unable to provide documents confirming that ethics approval was obtained for the study. After publication the corresponding author requested a retraction but did not provide any further details. Upon further investigation it was identified that some of the described tumour sizes exceed 12 mm—the maximum size allowed by the UK guidelines. The authors did not respond to any of the concerns raised.

None of the authors have responded to any correspondence from the editor/publisher about this retraction.

